# Sex Differences and Temporal Trends in Hospitalization for Catheter Ablation of Nonvalvular Atrial Fibrillation: A Single-Center Experience for 15 Years

**DOI:** 10.1155/2022/6522261

**Published:** 2022-07-04

**Authors:** Xiaodong Peng, Linling Li, Mengxia Zhang, Qianqian Zhao, Kui Wu, Rong Bai, Yanfei Ruan, Changsheng Ma, Nian Liu

**Affiliations:** ^1^Department of Cardiology, Beijing Anzhen Hospital, Capital Medical University, Beijing, China; ^2^National Clinical Research Center for Cardiovascular Diseases, Beijing, China

## Abstract

**Background:**

There exist sex differences in the clinical profile, management, and outcome of atrial fibrillation (AF). Catheter ablation of AF has become a first-line therapy and has markedly made headway over the recent decades. Little is known about sex differences and temporal trends in hospitalization for catheter ablation of AF in the real-world setting.

**Methods:**

We retrospectively retrieved medical records of patients at Beijing Anzhen Hospital between January 2005 and December 2019. The patients undergoing catheter ablation of AF were enrolled. Demographical and clinical data were compared between sexes. The temporal trends of sex differences were evaluated.

**Results:**

We identified 13502 male patients (66.8%) and 6713 female patients (33.2%). The number of patients undergoing AF ablation had remarkably increased over time, but no sex differences were observed (*p*=0.17). The median age of women was five years older than that of men (*p* < 0.001). The median time of in-hospital stay for the women decreased from 11 days to 4 days and for the men from 9 to 4 days. In-hospital mortality was 0.03% and 0.01% for women and men, respectively, with no significant difference between sexes. The women were more likely to have a comorbid diagnosis of hypertension and heart failure than men (*p* < 0.001). The CHA_2_DS_2_-VA score was higher in women than in men (1.64 vs. 1.28, *p* < 0.001). The temporal trend in the score increased in women from 1.17 to 1.81 (*p* < 0.001) and in men from 0.91 to 1.41 (*p* < 0.001). The percentage of patients with CHA_2_DS_2_-VA score ≥2 was higher in women than in men (49.8% vs. 35.8%, *p* < 0.001), and the temporal trend of this sex gap was nearly doubled (8.0% in 2005–2007 vs. 15.5% in 2017–2019, *p*=0.03).

**Conclusions:**

Safety of catheter ablation for AF was comparable in both sexes. In contrast, the women showed a higher CHA_2_DS_2_-VA score than men. The percentage of patients with CHA_2_DS_2_-VA score ≥2 increased more quickly in women than in men. Furthermore, sex-specific research is warranted to reduce this sex disparity.

## 1. Introduction

Atrial fibrillation (AF) is one of the most common arrhythmias globally. There are sex differences in AF across the course of the disease, from epidemiology and pathophysiological mechanisms to management and outcomes. The Framingham study showed that the age-adjusted incidence of AF was 49.4 per 1000 person-years and 96.2 per 1000 person-years in women and men, respectively [1]. Although the morbidity of AF is lower in women, the total number of women with AF is higher than that of men with AF. This is related to the greater longevity of women [2]. Other studies show that women are more vulnerable to severe complications associated with AF [3]. Meanwhile, it is more difficult for women than for men to maintain sinus rhythm by taking antiarrhythmic drugs [4]. There has been increasing attention to sex differences in the field. Determining gender differences using gender-specific data is a prerequisite resolving sex disparities.

AF catheter ablation as an advanced therapy has been the first-line therapy for patients with AF since it reduces symptom burden and improves the quality of life in patients. Several studies have demonstrated that there are sex gaps in the procedural complications and clinical outcomes. Usually, women have increased hospitalization rates after AF ablation and are more likely to have procedural complications. Few studies have focused on sex differences in referral for AF catheter ablation. Given that the advances in device technology and ablation strategies have significantly improved the efficiency and efficacy of the procedure in the recent decades, little is known about how these advances impact on sex differences in undergoing AF ablation in the real-world setting. Herein, we performed a retrospective study on consecutive patients undergoing AF ablation in the largest AF center in China and evaluated the sex difference and its temporal trend over 15 years.

## 2. Methods

### 2.1. Study Population

The study population was selected from Beijing Anzhen Hospital electronic medical records (EMRs) between January 2005 and December 2019. Those who failed or consumed intolerant antiarrhythmic drugs were admitted for AF ablation to improve symptoms of AF recurrences. We enrolled the patients by identifying the *International Classification of Disease, Tenth Revision, Clinical Modification* (*ICD-10-CM*) code for atrial fibrillation (I48.0–I48.2, I48.9). Inclusion criteria were patients aged ≥18 years who underwent AF catheter ablation. Patients were excluded if they had (1) surgical cardiac ablation or (2) valvular AF (mitral stenosis or valvular repair or replacement). The study and data collection were performed based on protocols approved by the ethics committee of Beijing Anzhen Hospital.

### 2.2. Clinical Characteristics

Demographic data (age and sex), length of hospital stay, and data on in-hospital deaths, comorbidities, and AF subtype (paroxysmal or nonparoxysmal) were collected. AF lasting less than seven days was considered as paroxysmal AF. If AF sustained beyond seven days, nonparoxysmal AF could be defined. Major comorbidities included hypertension, type 2 diabetes (T2D), heart failure (HF), stroke/transient ischemic attack (TIA)/thromboembolism (TE), and vascular diseases (prior myocardial infarction, peripheral artery disease, or aortic plaque) [5]. The CHA_2_DS_2_-VA score (excluding the sex category) was applied in this study. Female sex can be considered as a stroke risk modifier, rather than an overall risk factor [6].

### 2.3. Statistical Analyses

The patients were classified into two groups by sex. Their baseline characteristics and CHA_2_DS_2_-VA score were compared. Continuous data were presented as median (interquartile range, IQR) or mean ± standard deviation (SD). Categorical data were summarized as percentages. To further investigate the association between different sexes and hospital deaths, multivariate logistic regression was performed to calculate odds ratios (ORs) with 95% confidence intervals (CIs). Sex differences in age, time in hospital, and CHA_2_DS_2_-VA score were assessed using the Wilcoxon rank-sum test. Chi-square tests were applied to determine the statistical differences in categorical data. Temporal trends in categorical variables were assessed using Chi-square tests for association. The natural logarithmic transformation had been performed for the difference in the proportion of high CHA_2_DS_2_-VA scores (≥2) between women and men. Then, the score difference was assessed by ANOVA trend analyses. The one-way ANOVA with a polynomial contrast procedure test was performed to identify temporal differences in continuous variables. Statistical significance was defined as a two-sided *p* value <0.05, and all statistical analyses were performed using SPSS 19.0 software (IBM Corporation, New York, USA).

## 3. Results

### 3.1. Demographic Features, Hospital Stay Time, and In-Hospital Mortality

**Figure 1:**
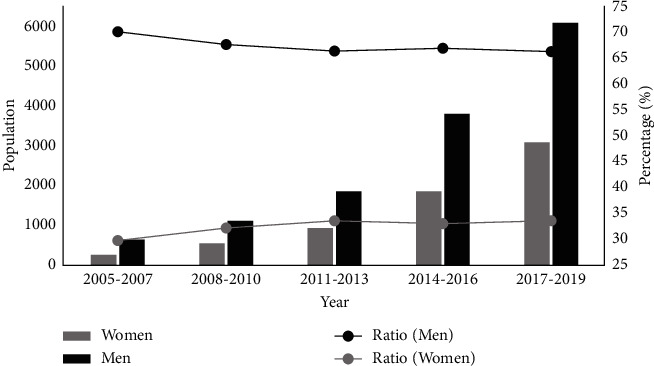
Trends in the number and sex ratio of patients undergoing catheter ablation for atrial fibrillation according to sex.

In this survey, a total of 20215 patients with nonvalvular AF undergoing catheter ablation were enrolled, including 13502 men (66.8%) and 6713 women (33.2%). The number of men and women significantly increased over time (from 601 in 2005–2007 to 6087 in 2017–2019 and 254 in 2005–2007 to 3094 in 2017–2019, respectively) (Figure 1); the temporal trend in male : female ratio did not change significantly throughout the study period (*p*=0.17, Table 1). The median age of women was 64 years, and men were five years younger than women. The age gap between sexes decreased from 7 years in 2005–2007 to 5 years in 2017–2019, but no statistical significance was found in the trend of the age gap between sexes (*p*=0.4) (Table 2). The proportion of elderly patients (≥65 years) in women (49.8%) was remarkably higher than that of men (29.5%) (*p* < 0.001), and an increasing trend in the proportion of women aged ≥65 years was noted, but not in that of men. The median time of hospital stay decreased from 11 to 4 days in women and from 9 days to 4 days in men. In-hospital mortality was 0.01% and 0.03% for the men and women, respectively, which was not statistically different (OR 1.796, 95% CI: 0.227–14.187, *p*=0.579).

### 3.2. Patterns of Atrial Fibrillation and Ablation

Paroxysmal AF was more frequent in women than in men (69.0% vs. 56.2%, *p* < 0.001). No statistical significance was noted in the redo procedure between women and men (13.2% vs. 13.1%, *p*=0.837). The proportion of patients undergoing cryoablation was similar between men and women (1.3% vs. 1.1%, *p*=0.252).

### 3.3. Comorbidities of Atrial Fibrillation between Sexes

Hypertension was the most common comorbidity in patients. The percentage of patients with hypertension was higher in women than in men (58.1% vs. 48.5%, *p* < 0.001). We observed a significant increase in the rate of hypertension in men and women over time. Compared with men, women were more likely to have a comorbid diagnosis of heart failure, hyperlipemia, congenital heart disease, hyperthyroidism, hypothyroidism, and cancer (*p* < 0.05) (Table 1). Dilated cardiomyopathy (DCM) was more frequent in male patients (0.6% vs. 0.1%) (*p* < 0.001). The incidence of thromboembolic events such as stroke and TIA were comparable between men and women (*p*=0.524). Increased temporal trends of heart failure, hypertension, stroke, and vascular diseases were observed in both sexes (Table 2).

### 3.4. The Temporal Trends of CHA_2_DS_2_-VA Score between Sexes

**Figure 2:**
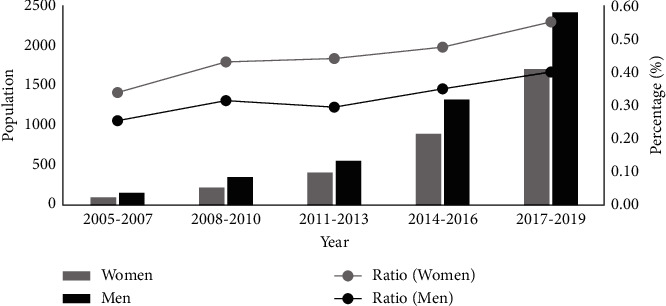
Temporal trends of the CHA_2_DS_2_-VA score ≥ 2 in the population and its proportion within women and men.

**Figure 3:**
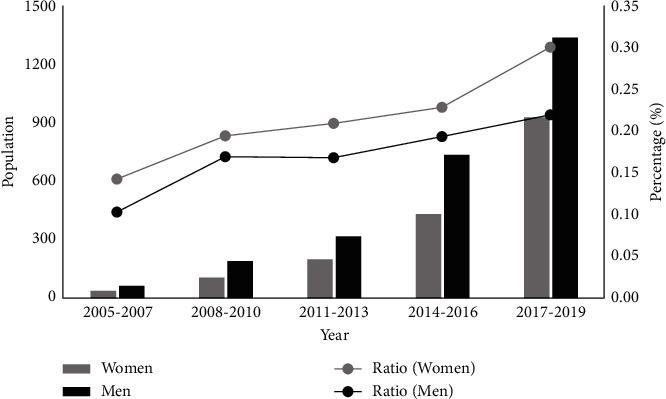
Temporal trends of the CHADS_2_ score ≥ 2 in the population and its proportion within women and men.

The CHA_2_DS_2_-VA score was higher in women than in men (1.64 vs. 1.28, *p* < 0.001). The temporal trend in the score increased in women from 1.17 to 1.81 (*p* < 0.001) and in men from 0.91 to 1.41 (*p* < 0.001); the sex gap in the CHA_2_DS_2_-VA score did not narrow over time. The percentage of patients with the CHA_2_DS_2_-VA score ≥2 was higher in women than in men (49.8% vs. 35.8%, *p* < 0.001), and the temporal trend of this sex gap was enlarged (8.0% in 2005–2007 vs. 15.5% in 2017–2019, *p*=0.03, Figure 2). The same results were observed when comparing sex discrepancies by the CHADS_2_ score (Figure 3).

## 4. Discussion

In this retrospective study with a large cohort of 20215 cases from a single center, we evaluated the sex differences and temporal trends in hospitalization for catheter ablation of nonvalvular atrial fibrillation for 15 years. We observed that (1) the number of women was almost half of that of men, the number of men and women significantly increased over time, and the temporal trend in the man/woman ratio did not change substantially; (2) the median time of in-hospital stay and the in-hospital mortality were comparable between women and men; (3) women were five years older than men and had more comorbidities; and (4) the CHA_2_DS_2_-VA score was higher in women than in men. The percentage of patients with the CHA_2_DS_2_-VA score ≥2 increased more quickly in women than in men.

Sex differences in AF ablation have gained increasing interest. The safety of AF ablation is always the greatest concern for the sex difference. Several studies demonstrated that women were more likely to have a procedural complication than men. Kaiser et al. reported that women had a high risk of vascular complication, hemorrhage, and cardiac tamponade compared to men during the procedure [7]. Data from the Chinese Atrial Fibrillation Registry also showed that women had more vascular complications [8]. In the present study, we focused on in-hospital mortality, which was 0.01% and 0.03% for men and the women, respectively, that did not significantly change during the study period. An initial study by Cappato et al. showed a risk of AF ablation-related mortality of 1 per 1000 patients [9]. In a real-world setting, Deshmukh et al. reported that the in-hospital mortality of AF ablation for all US hospitals was 0.42% between 2000 and 2010 [10]. However, these two studies did not provide sex-specific mortality rates. In-hospital mortality in the present study is much lower than in previous studies. It is well known that the complications and the mortality of AF ablation are associated with the operator's experience and hospital volume. In line with this issue, data from Cleveland Clinic showed zero procedure-related deaths over 16 years [11]. Therefore, our data may be representative of experienced AF ablation centers in the real world. Intriguingly, the in-hospital stay time was gradually shortened during the study period for both sexes. In 2019, the average length of in-hospital stay was 4.3 ± 2.4 and 4.5 ± 2.6 days for the men and the women, respectively. AF ablation is the most common complicated ablation procedure, and there are challenging issues related to preprocedural, periprocedural, and postprocedural management. The progress of ablation device technology and ablation strategies, as well as the operator's experience, has substantially enhanced the safety, efficacy, and efficiency of AF ablation. Consequently, the life-threatening complications, procedural duration, and in-hospital stay time have been significantly improved. Inexperienced AF centers, even same-day discharge after AF ablation is feasible in the majority of patients [12]. Despite the lack of data about life-threatening complications of AF ablation in the present study, the same in-hospital mortality and the same hospital stay time between men and women suggested that excellent safety during the ablation procedure was indiscriminately provided for both sexes.

AF ablation has been the primary clinical service in many arrhythmia centers. The number of AF ablation cases has substantially increased in recent decades. Sharp increases in cases in the present study were noted in both sexes, and the temporal trend in the man/woman ratio did not significantly change. Our data were consistent with a nationwide cohort study from Denmark, in which the number of patients undergoing AF ablation almost tripled from 2005 to 2014, the majority of the patients were men, and the man/woman ratio remained constant over the study duration [13]. A study using Quebec administrative databases also demonstrated that the patients with AF undergoing AF ablation increased almost seven-fold in 10 years and the annual proportion of women in the AF ablation cohort had not surpassed 30% [14]. All the data support the finding that there has been no increase in the relatively low percentage of women undergoing AF ablation despite expansion of the uptake of AF ablation. Two large cross-sectional studies of Chinese cohorts reported that age-adjusted prevalence of AF was similar in women and men, implying that women with AF are less likely to be referred for the advanced therapy of ablation than men in China [15, 16].

For the direct comparison of the disease severity and complexity between men and women, the CHA_2_DS_2_-VA score (excluding the female sex) was used in the present study. Our data showed that this score was higher in women than in men and more women had scored ≥2 than men. Older age and more comorbidities in women accounted for this sex gap. Our study is consistent with previous studies. For instance, a study from Europe showed that the women undergoing catheter ablation for drug-refractory AF were older, had a long history of AF, and were more likely to have hypertension and valvular disease [17]. Intriguingly, the CHA_2_DS_2_-VA score increased in both sexes from 2005 to 2019 in the present study. An epidemiological study from South Korea showed that there was a significant increase in the proportion of high CHA_2_DS_2_-VASc scores (≥2) in patients with AF from 2008 to 2015 [18]. There are no data on CHA_2_DS_2_-VASc score trends in China. Since the prevalence of AF in East Asia is similar to and lower than that in Western countries, we speculated that the temporal trend in the CHA_2_DS_2_-VA score in the present study is unlikely to reflect the score shifting in the general AF population. The expansion of indication for AF ablation in the recent decade and improvements in the operator's experience are attributable to the temporal trend in this score.

Moreover, the temporal trend in the sex gap of the CHA_2_DS_2_-VA score did not narrow and instead showed an increasing tendency. Especially, in the patients with the CHA_2_DS_2_-VA score ≥2, our data clearly showed that this sex gap became wider over time, suggesting a decrease in sex disparity in AF ablation over time. To the best of our knowledge, no previous study investigated this issue. In a study of questionnaire measures, women often reported greater AF severity, frequency, and burden than men [19]. Consequently, female AF patients tended to be more symptomatic and may have been more likely to seek medical attention as a result. A study by Bhave et al. showed that the sex disparity in AF management resulted in fewer referrals for advanced therapies in women [20]. Previous work has suggested that fewer women access new technology services, such as smaller devices and less invasive procedures. For example, women were also less likely to undergo cardiac catheterization, percutaneous transluminal coronary angioplasty, coronary artery bypass surgery, pacemaker, and defibrillator implantation, than men [21–23]. Hence, the apparent impact of sex disparity is that women who do undergo AF often have more comorbidities and older age. Despite the finding that safety of catheter ablation for AF in the hospital was comparable in women and men in the present study, less favorable outcomes of AF ablation have been reported in women, such as more AF recurrence and more rehospitalization after the procedure. Compelling evidence demonstrates that earlier AF detection and ablation enhance efficacy [24, 25]. We speculate that the sex differences in hospitalization for AF ablation are attributable to the adverse clinical outcomes in women. The temporal trends of large gaps between sexes in the hospitalization for AF ablation raise a warning flag to resolve this issue.

The reasons for the sex differences in the hospitalization for AF ablation remain unknown. Sex-related differences of biology or physiology cannot explain the sex differences observed in the present study. Socioeconomic inequalities between women and men need to be taken into account [26]. The sex inequalities in individual incomes and education levels likely greatly contribute to this issue. Furthermore, investigation to determine the reasons for the sex differences in the hospitalization for AF ablation is warranted.

The study has several limitations. First, it is a study from one of the largest arrhythmia centers in China and is therefore limited by selection bias. The conclusion may be different from those in local hospitals with small volumes or other regions in China. However, it may represent a developing trend of AF ablation in China. Second, the information about periprocedural complications and the follow-up is lacking. We cannot further evaluate the differences and trends in the safety and efficacy of AF catheter ablation between women and men. Third, the electronic medical records do not include the socioeconomic information like educational level and income, which may contribute to the gender difference observed in our study.

## 5. Conclusions

The present study evaluated the sex differences and temporal trends of hospitalization for catheter ablation of AF from one of the largest arrhythmia centers in China. Safety of catheter ablation for AF was comparable between women and men. However, the women showed a higher CHA_2_DS_2_-VA score than the men. The percentage of patients with CHA_2_DS_2_-VA score ≥2 increased more quickly in women than in men. The reasons for sex differences in the hospitalization for AF ablation remain unknown. Furthermore, sex-specific research is warranted to solve this issue.

## Figures and Tables

**Table 1:** Baseline characteristics of AF patients by gender.

Characteristics	Women (*n* = 6713)	Men (*n* = 13502)	*p* value
Age, median (IQR), y	64 (58–70)	59 (51–66)	<0.001
<65, *n* (%)	3373 (50.25)	9517 (70.5)	<0.001
65–74, *n* (%)	2561 (38.15)	3128 (23.2)	<0.001
≥75, *n* (%)	779 (11.60)	857 (6.3)	<0.001
Gender constituent ratio (%)	33.2	66.8	<0.001
Time in-hospital, median (IQR), d	5 (3–7)	5 (3–6)	<0.001
Health insurance, *n* (%)	6353 (94.6)	12744 (94.4)	0.494
Marital status			
Married, *n* (%)	6317 (94.1)	13164 (97.5)	<0.001
Unmarried/divorced/widowed, *n* (%)	396 (5.9)	338 (2.5)	
Hospital deaths, *n* (%)	2 (0.03)	2 (0.01)	0.476
Paroxysmal AF, *n* (%)	4632 (69.0%)	7583 (56.2%)	<0.001
Persistent AF, *n* (%)	1645 (24.5%)	4918 (36.4%)	<0.001
Previous catheter ablation, *n* (%)	884 (13.2%)	1764 (13.1%)	0.837
Cryoablation, *n* (%)	88 (1.3%)	152 (1.1%)	0.252
Comorbidity, *n* (%)			
Heart failure	242 (3.6%)	345 (2.6%)	<0.001
Hypertension	3899 (58.1%)	6551 (48.5%)	<0.001
Diabetes mellitus	1079 (16.1%)	2113 (15.6%)	0.436
Stroke/TIA/TE	415 (6.2%)	866 (6.4%)	0.524
Vascular disease	837 (12.5%)	1814 (13.4%)	0.055
Hyperlipemia	1789 (26.6%)	3316 (24.6%)	0.001
Coronary heart disease	1026 (15.3%)	2100 (15.6%)	0.618
Cardiomyopathy	129 (1.9%)	361 (2.7%)	0.001
HCM	120 (1.8%)	240 (1.8%)	0.959
DCM	7 (0.1%)	86 (0.6%)	<0.001
Congenital heart disease	36 (0.5%)	40 (0.3%)	0.009
Hyperthyroidism	90 (1.3%)	129 (1.0%)	0.013
Hypothyroidism	317 (4.7%)	249 (1.8%)	<0.001
Malignant tumor	42 (0.6%)	38 (0.3%)	<0.001
PAH	24 (0.4%)	29 (0.2%)	0.062
CHA_2_DS_2_-VA score	1.64 ± 1.33	1.28 ± 1.26	<0.001
Score = 0	1560 (23.2%)	4406 (32.6%)	<0.001
Score = 1	1811 (27.0%)	4264 (31.6%)	<0.001
Score ≥ 2	3342 (49.8%)	4832 (35.8%)	<0.001
CHADS_2_ score	1.00 ± 0.93	0.85 ± 0.91	<0.001
Score = 0	2204 (32.8%)	5521 (40.9%)	<0.001
Score = 1	2814 (41.9%)	5344 (39.6%)	0.001
Score ≥ 2	1695 (25.2%)	2637 (19.5%)	<0.001

**Table 2:** Temporal trends in clinical features of women and men.

	2005–2007	2008–2010	2011–2013	2014–2016	2017–2019	Linear *χ*^2^	*p* value
Women							
AF patients, *n*	254	536	949	1880	3094		
Age, median (IQR)	62 (55–67)	63 (56–69)	63 (57–69)	64 (58–70)	65 (59–71)	—	<0.001
Age ≥ 75, *n* (%)	8 (3.1)	35 (6.5)	77 (8.1)	239 (12.7)	420 (13.6)	52.2	<0.001
Age 65–74, *n* (%)	90 (35.4)	207 (38.6)	334 (35.2)	676(36.0)	1254 (40.5)	6.9	0.009
Time in hospital (d; median, IQR)	11 (8–13)	9 (7–11)	6 (4–8)	5 (3–6)	4 (3–5)	—	<0.001
Hospital deaths, *n* (%)	—	—	—	—	2 (0.06%)	—	0.230
Comorbidity, *n* (%)							
Heart failure	6 (2.4)	7 (1.3)	9 (0.9)	44 (2.3)	176 (5.7)	52.1	<0.001
Hypertension	121 (47.6)	301 (56.2)	540 (56.9)	1077 (57.3)	1860 (60.1)	14.8	<0.001
Diabetes mellitus	32 (12.6)	83 (15.5)	164 (17.3)	267 (14.2)	533 (17.2)	2.8	0.09
Stroke/TIA/TE	7 (2.8)	32 (6.0)	55 (5.8)	85 (4.5)	236 (7.6)	12.0	0.001
Vascular disease	20 (7.9)	41 (7.6)	80 (8.4)	232 (12.3)	464 (15.0)	44.7	<0.001
CHA_2_DS_2_-VA score (mean ± SD)	1.17 ± 1.06	1.43 ± 1.19	1.46 ± 1.26	1.56 ± 1.31	1.81 ± 1.39	—	<0.001
Score 0, *n* (%)	78 (30.7)	128 (23.9)	246 (25.9)	479 (25.5)	629 (20.3)	21.3	<0.001
Score 1 *n* (%)	91 (35.8)	177 (33.0)	286 (30.2)	506 (26.9)	751 (24.3)	36.3	<0.001
Score ≥ 2 *n* (%)	85 (33.5)	231 (43.1)	417 (43.9)	895 (47.6)	1714 (55.4)	85.5	<0.001
CHADS_2_ score (mean ± SD)	0.74 ± 0.74	0.89 ± 0.84	0.93 ± 0.88	0.95 ± 0.90	1.11 ± 0.97	—	<0.001
Score 0, *n* (%)	106 (41.7)	191 (35.6)	331 (34.9)	653 (34.7)	923 (30.0)	23.7	<0.001
Score 1, *n* (%)	112 (44.1)	241 (45.0)	420 (44.3)	798 (42.4)	1243 (40.2)	7.6	0.006
Score ≥ 2, *n* (%)	36 (14.2)	104 (19.4)	198 (20.9)	429 (22.8)	928 (30.0)	70.5	<0.001
Men							
AF patients, *n*	601	1121	1881	3812	6087		
Age, median (IQR)	55 (47–65)	57 (48–65)	58 (50–65)	59 (51–66)	60 (52–66)	—	<0.001
Age ≥ 75, *n* (%)	33 (5.5)	56 (5.0)	104 (5.5)	261 (6.8)	403 (6.6)	5.7	0.017
Age 65–74, *n* (%)	124 (20.6)	247 (22.0)	377 (20.0)	826 (21.7)	1554 (25.5)	23.6	<0.001
Time in hospital (d; median, IQR)	9 (7–12)	8 (6–10)	6 (4–7)	4 (3–6)	4 (3–5)	—	<0.001
Hospital deaths, *n* (%)	—	1 (0.09%)	—	—	1 (0.02%)	—	0.529
Comorbidity, *n* (%)							
Heart failure	6 (1.0)	13 (1.2)	14 (0.7)	75 (2.0)	237 (3.9)	68.6	<0.001
Hypertension	223 (37.1)	513 (45.8)	799 (42.5)	1870 (49.1)	3146 (51.7)	73.8	<0.001
Diabetes mellitus	54 (9.0)	147 (13.1)	238 (12.7)	624 (16.4)	1050 (17.2)	46.4	<0.001
Stroke/TIA/TE	18 (3.0)	72 (6.4)	148 (7.9)	181 (4.7)	447 (7.3)	7.7	0.006
Vascular disease	37 (6.2)	102 (9.1)	169 (9.0)	514 (13.5)	992 (16.3)	113.9	<0.001
CHA_2_DS_2_-VA score (mean ± SD)	0.91 ± 1.08	1.14 ± 1.20	1.11 ± 1.19	1.25 ± 1.24	1.41 ± 1.30	—	<0.001
Score 0, *n* (%)	277 (46.1)	421 (37.6)	711(37.8)	1280(33.6)	1717(28.2)	131.9	<0.001
Score 1, *n* (%)	171 (28.5)	347 (28.4)	613 (32.6)	1193(31.3)	1940(31.9)	4.4	0.035
Score ≥ 2, *n* (%)	153 (25.5)	353 (28.9)	557 (29.6)	1339(35.1)	2430(39.9)	124.6	<0.001
CHADS_2_ score (mean ± SD)	0.61 ± 0.78	0.78 ± 0.88	0.76 ± 0.91	0.84 ± 0.88	0.93 ± 0.93	—	<0.001
Score 0, *n* (%)	321 (53.4)	506 (45.1)	895 (47.6)	1558 (40.9)	2241 (36.8)	111.8	<0.001
Score 1, *n* (%)	218 (36.3)	425 (37.9)	670 (35.6)	1519 (39.8)	2512 (41.3)	17.6	<0.001
Score ≥ 2, *n* (%)	62 (10.3)	190 (16.9)	316 (16.8)	735 (19.3)	1334 (21.9)	63.0	<0.001

## Data Availability

The dataset analyzed during the current study is available from the corresponding author on reasonable request.
